# IDH mutant high-grade gliomas

**DOI:** 10.3389/fnmol.2025.1662414

**Published:** 2025-08-29

**Authors:** Santosh Valvi, Maryam Fouladi, Michael J. Fisher, Nicholas G. Gottardo

**Affiliations:** ^1^Department of Pediatric and Adolescent Oncology and Hematology, Perth Children’s Hospital, Perth, WA, Australia; ^2^Brain Tumor Research Program, The Kids Research Institute Australia, Perth, WA, Australia; ^3^Division of Pediatrics, School of Medicine, The University of Western Australia, Perth, WA, Australia; ^4^Department of Pediatrics, Nationwide Children’s Hospital, Columbus, OH, United States; ^5^Division of Oncology, Children’s Hospital of Philadelphia, Philadelphia, PA, United States; ^6^Department of Pediatrics, University of Pennsylvania Perelman School of Medicine, Philadelphia, PA, United States

**Keywords:** IDH mutation, high-grade glioma, IDH inhibitors, clinical trials, pediatric, adolescent and young adults

## Abstract

Gliomas are the most common type of malignant primary central nervous system (CNS) tumors, resulting in significant morbidity and mortality in children and adolescent and young adult (AYA) patients. The discovery of mutations in isocitrate dehydrogenase (IDH) genes has dramatically changed the classification and understanding of gliomas. IDH mutant gliomas have distinct clinical, pathological, and molecular features including a favorable prognosis and response to therapy compared to their wildtype counterparts. Although more common in adults, 5–15% of pediatric gliomas have IDH mutations. In this review, we provide a comprehensive summary of the current knowledge on IDH mutant high-grade gliomas (HGG), including their biology, clinical features, diagnosis, treatment, and prognosis. We also discuss future directions in research and clinical management with particular attention to the AYA cohort.

## 1 Introduction

Gliomas are a heterogeneous group of tumors that arise from glial progenitor cells in the central nervous system (CNS) (Sanai et al., 2005). They comprise approximately 30% of all CNS tumors in children and adolescent and young adult (AYA) population leading to substantial morbidity and mortality (Yamasaki, 2022; Ostrom et al., 2019). IDH mutant gliomas are characterized by mutations in the IDH genes more commonly found in lower-grade gliomas and secondary glioblastoma. Historically, gliomas have been classified based on histological features; however, the recent advances in cancer genomics have enabled integration of molecular profiles into their diagnosis (Louis et al., 2021). This is reflected in the 2021 World Health Organization (WHO) Classification of Tumors of the CNS which categorizes gliomas, glioneuronal tumors, and neuronal tumors into six groups: adult-type diffuse gliomas, pediatric-type diffuse low-grade gliomas (LGG), pediatric-type diffuse high-grade gliomas (HGG), circumscribed astrocytic gliomas, glioneuronal and neuronal tumors, and ependymal tumors. Adult-type diffuse gliomas are further subclassified into astrocytoma (IDH mutant, CNS WHO Grades 2–4), oligodendroglioma (IDH mutant and 1p/19q-codeleted, WHO Grades 2 and 3), and glioblastoma (IDH wildtype) (Louis et al., 2021). The presence of 1p/19q codeletion along with IDH mutation is a key diagnostic and prognostic factor. IDH mutant and 1p/19q-codeleted gliomas demonstrate increased sensitivity to chemotherapy, especially alkylating agents and are associated with prolonged survival when treated with a combination of radiation and chemotherapy. This molecular profile confers a more favorable prognosis compared to gliomas lacking these genetic alterations.

Isocitrate dehydrogenase (IDH) enzymes play critical roles in distinct cellular metabolic pathways (Koh et al., 2004; Tang et al., 2022). There are three isoforms of IDH enzymes with IDH1 or IDH2 gene mutations leading to aberrant enzyme activity and disruption in redox balance (Montesinos et al., 2022; Liu et al., 2020; Dang and Su, 2017). IDH mutations are prevalent in several malignancies including myeloid leukemias and solid tumors (DiNardo et al., 2015; Paschka et al., 2010; Mardis et al., 2009; Watts et al., 2023). Their association with gliomas was first reported in 2008 (Parsons et al., 2008) and their presence is now a defining feature of diffuse gliomas with therapeutic and prognostic implications (Hartmann et al., 2009; De Carli et al., 2009; Reitman and Yan, 2010). IDH mutations are seen in 80% of grade 2 and 3 diffuse gliomas. Glioblastoma are the most common malignant brain tumors which can develop rapidly (primary or *de novo* glioblastoma) or slowly through transformation of a lower-grade glioma (secondary glioblastoma). Although Hans-Joachim made the distinction between primary and secondary glioblastomas in 1940, these tumors are histopathologically indistinguishable. However, they constitute biologically distinct entities. They affect patient at different ages with divergent triggering genetic events, exhibit distinct proteomic and transcriptomic profiles and display differing responses to radiation and chemotherapy. IDH mutations are seen in 73% of secondary glioblastomas but only 3.7% of primary glioblastoma suggesting malignant transformation of lower grade IDH mutant astrocytoma and oligodendroglioma (Han et al., 2020). Due their higher prevalence, IDH mutations are a highly selective molecular marker of secondary glioblastoma. Tremendous advances have been made in the understanding of IDH mutant gliomas (Bennett et al., 2023). For low grade IDH mutant gliomas the treatment paradigm has shifted from the traditional approach of surgery followed by active surveillance or adjuvant radiation and/or chemotherapy based on recurrence risk, to the incorporation of IDH inhibitors (Mohile et al., 2022; Weller et al., 2021; Miller et al., 2023). IDH-mutant gliomas are relatively uncommon in pediatric and adolescent/young adult (AYA) populations (Pollack et al., 2011; Yeo et al., 2023) and consequently, standardized management guidelines akin to those established for adults are currently lacking.

This comprehensive review begins by outlining the fundamental biology of IDH enzymes including their role in the normal cellular metabolism. It then explores the spectrum of IDH mutations and their downstream effects on cellular pathways. The next section provides insights into the molecular and clinical biology of IDH mutant gliomas detailing their classification, epidemiology, co-occurring genetic alterations, clonal evolution and malignant transformation of LGG into HGG. Special attention is given to the disease’s impact on pediatric and AYA populations, as well as the unique entity of Primary Mismatch Repair-Deficient IDH-mutant Astrocytoma (PMMRDIA). Section 4 reviews the clinical features and diagnostic workup, while Section 5 describes histopathological and molecular diagnostic approaches, including DNA methylation profiling and the application of machine learning algorithms. Section 6 discusses the current diagnostic platforms and classification frameworks. Ection 7 summarizes existing and emerging therapeutic strategies, encompassing preclinical research and ongoing clinical trials. This is followed by an overview of survival outcomes and prognostic biomarkers. The review concludes with a discussion on future directions, emphasizing the unmet clinical and research needs in pediatric and AYA patient populations.

## 2 Biology of IDH enzymes

### 2.1 IDH enzymes

**Figure 1:**
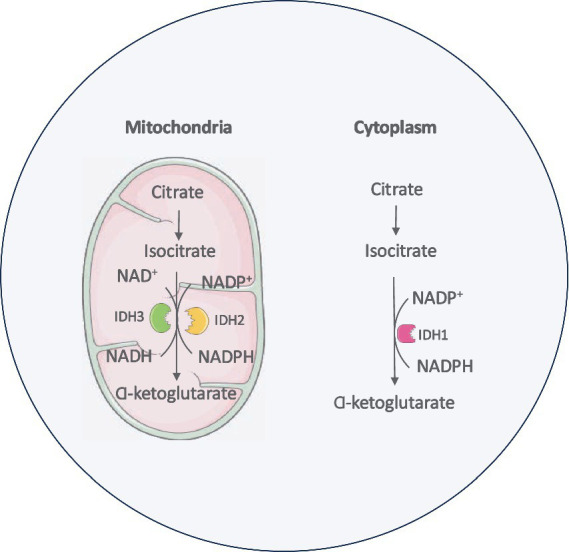
IDH enzyme isoforms and metabolic pathway.

There are three isoforms of IDH enzymes that are essential in several major cellular metabolic processes, such as the Krebs cycle, glutamine metabolism, lipogenesis and redox regulation (Koh et al., 2004). IDH1, IDH2 and IDH3 are involved in the central metabolic regulation and are encoded by different genes (Liu et al., 2020; Dang and Su, 2017; Kaminska et al., 2019). These isoforms have distinct cellular localizations with IDH1 located in the cytoplasm and IDH2 and IDH3 in the mitochondria (Reitman and Yan, 2010). IDH enzymes are central to the metabolic oxidative reactions involved in the Krebs cycle (Tang et al., 2022). Their catalytic sites display affinity toward isocitrate. IDH1 and IDH2 enzymes use nicotinamide adenine dinucleotide phosphate (NADP) and a divalent metal cation like magnesium or manganese as cofactors to catalyze the conversion of isocitrate to *α*-ketoglutarate (α-KG) and NADPH. The holoenzyme IDH3 localized to the mitochondria catalyzes nicotinamide adenine dinucleotide (NAD)-dependent α-KG production (Liu et al., 2020; Kaminska et al., 2019; Abla et al., 2020) (Figure 1).

α-Ketoglutarate (α-KG) is a key intermediate of the Krebs and is essential for cellular energy metabolism. Beyond its role in oxidative phosphorylation, α-KG serves as a critical cofactor for 2-oxoglutarate-dependent dioxygenases, a class of enzymes involved in diverse biological processes, including epigenetic regulation, collagen biosynthesis, and fatty acid metabolism (Kaminska et al., 2019; Miller et al., 2017; Vatrinet et al., 2017). NADPH plays a vital role in maintaining cellular redox homeostasis and defending against oxidative stress induced by reactive oxygen species (ROS). It provides reducing equivalents required for the regeneration of antioxidant systems, including the peroxiredoxin–thioredoxin pathway, which is essential for the detoxification of ROS and the preservation of cellular integrity (Dang and Su, 2017; Kil et al., 2007; Minard and McAlister-Henn, 1999; Yoo et al., 2020). In the brain, a substantial proportion of cellular NADPH is generated by IDH enzymes, particularly the cytosolic and mitochondrial isoforms. This NADPH production is critical for supporting various metabolic pathways in the brain, including lipid biosynthesis and maintenance of redox balance, both of which are essential for normal neuronal function and brain homeostasis (Bleeker et al., 2010).

**Figure 2:**
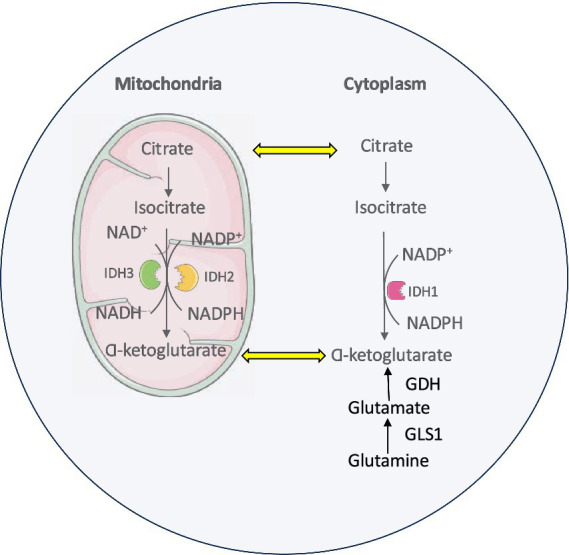
Glutamine metabolic pathway and interaction with IDH enzymes in cancer cell.

Glutamine serves as a key Krebs cycle substrate in proliferating cancer cells. It is one of the most abundant nonessential amino acids in circulation supporting all major biosynthetic processes in rapidly dividing cells like cancer. Through glutaminolysis, glutamine is metabolized to *α*-KG which is further catalyzed into isocitrate in the cytoplasm. After getting imported into mitochondria, IDH2 oxidizes this isocitrate back into *α*-KG (Hayes et al., 2020). This glutamine metabolic pathway supports the sustained production of nucleotides, lipids, and other essential macromolecules necessary for cancer cell proliferation and survival as well as provides reducing equivalents to fortify mitochondrial ROS defenses (Yoo et al., 2020; Metallo et al., 2011) (Figure 2).

### 2.2 IDH mutations

**Figure 3:**
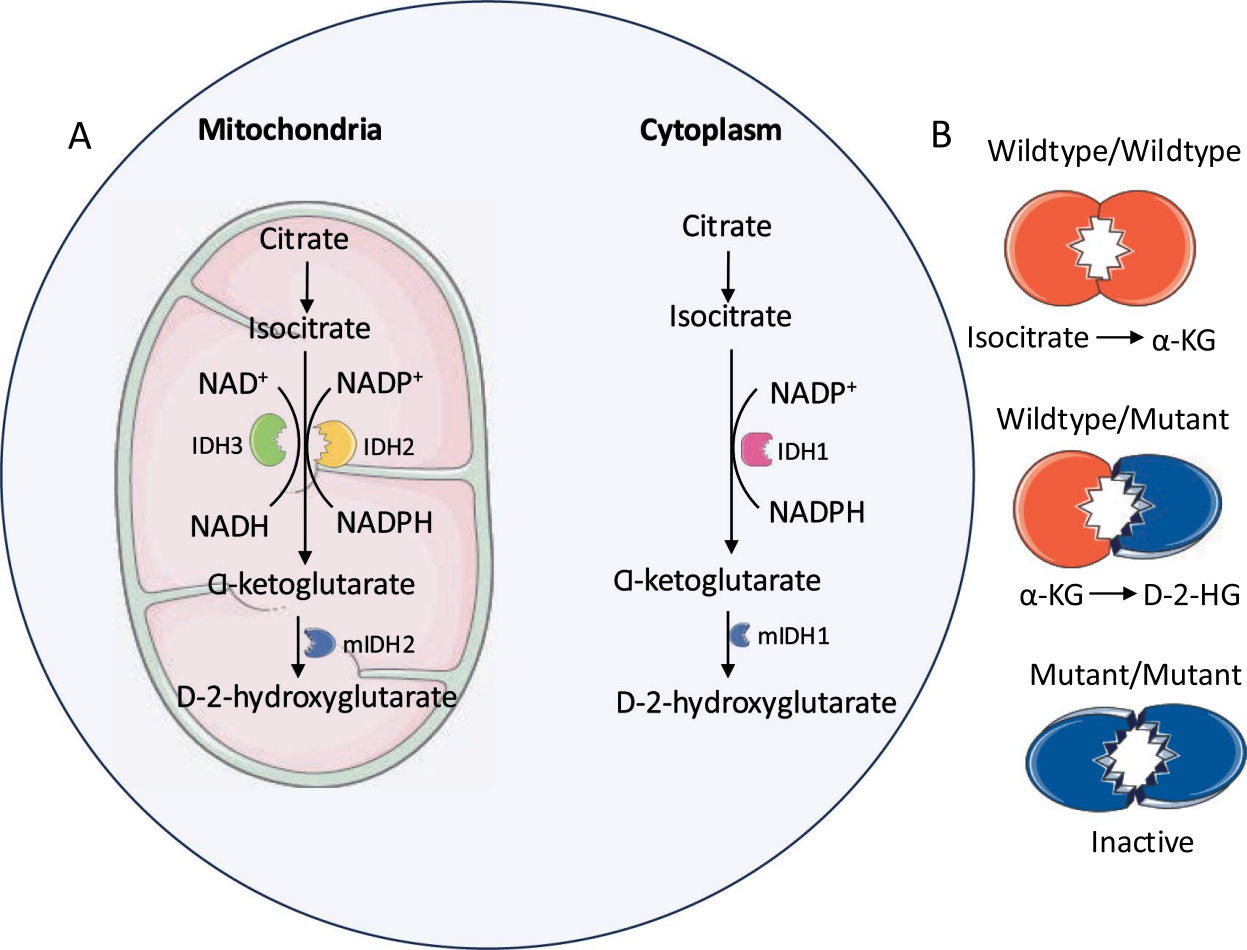
IDH mutations: **(A)** Mutant IDH1 (cytoplasm) and IDH2 (mitochondria) enzymes covert *α*-KG to D-2-HG. **(B)** Mutant IDH enzymatic activity requires wildtype/mutant heterodimer.

IDH mutations lead to the development of different types of cancers. The missense and heterozygous cancer-associated mutations typically occur at Arginine 132 in IDH1 and Arginine 172 in IDH2, impairing the ability of mutant IDH to bind with isocitrate (Yan et al., 2009; Pirozzi and Yan, 2021). There are no reported IDH3 mutations associated with cancer (Kaminska et al., 2019; Reitman et al., 2011). The IDH heterodimers contain a version of wildtype IDH1 and a version with the R132H mutation (Figure 3). This heterodimerization leads to substrate channeling (Ward et al., 2013; Pietrak et al., 2011) where the IDH1 wild-type component of the dimer converts isocitrate into *α*-KG to produce NADPH, whereas the mutant part of the dimer acquires neomorphic activity, promoting the conversion of α-KG to D-2-hydroxyglutarate (D-2-HG), which accumulates to very high levels, 10-to 100-fold higher than in IDH wildtype cells (Tang et al., 2022; Montesinos et al., 2022; Liu et al., 2020; Dang et al., 2009).

### 2.3 Effects of IDH mutations

**Figure 4:**
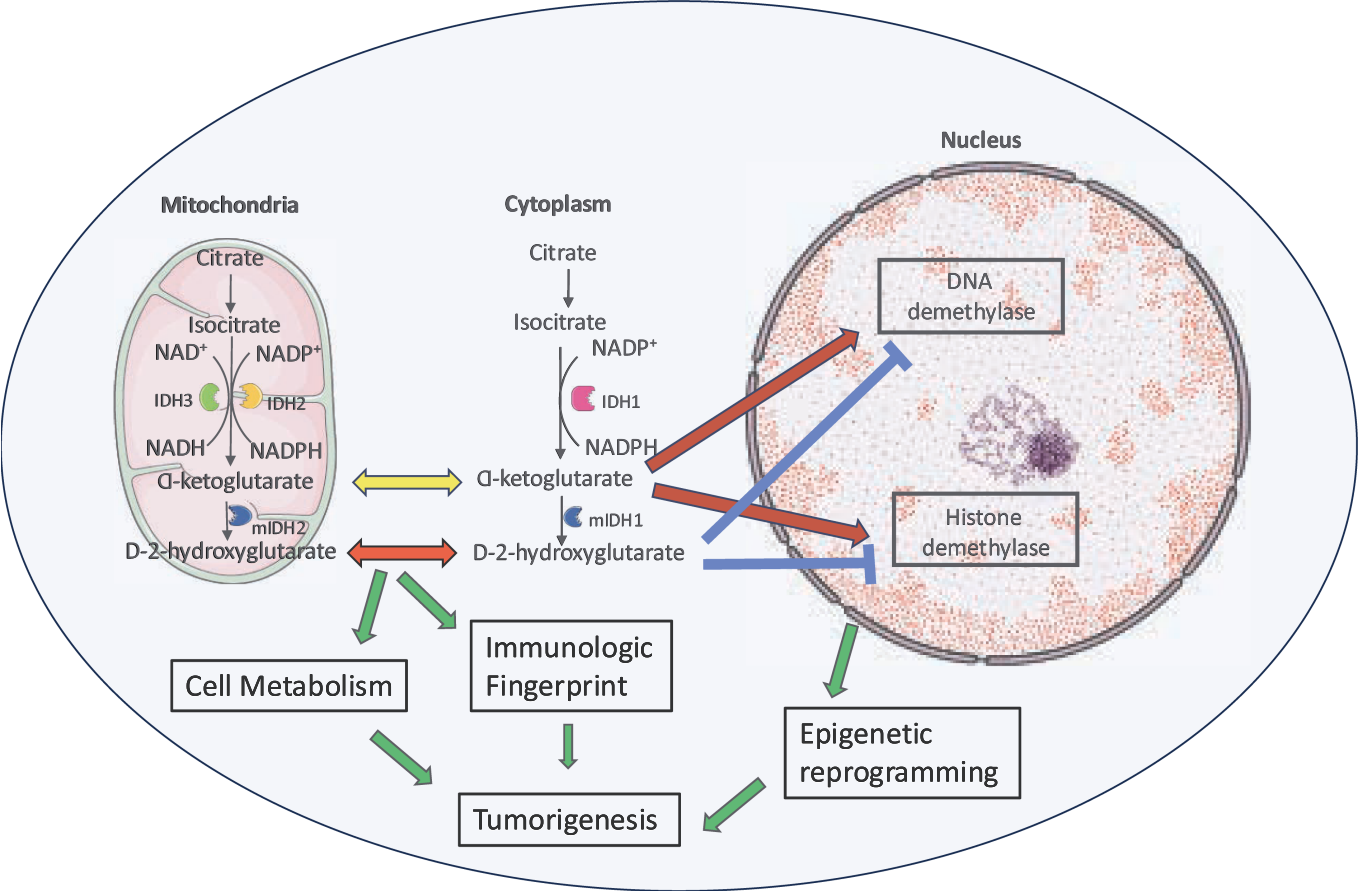
Role of D-2-HG in tumorigenesis. Increased levels of D-2-HG result in cell growth by histone and DNA hypermethylation, interference with normal cellular metabolic pathways and effect on tumor microenvironment.

Mutant IDH enzyme leads to tumorigenesis by a variety of mechanisms (Figure 4). Oncometabolite D-2-HG is structurally similar to α-KG and has an effect on cellular metabolism, cancer biology and oncogenesis (Prensner and Chinnaiyan, 2011). It has been shown to be elevated in glioma cells but not in the normal brain cells (Xu et al., 2011).

#### 2.3.1 Increased DNA and histone methylation

D-2-HG inhibits the α-KG dependent dioxygenase family including ten-eleven translocation (TET) DNA modifying enzymes and jumonji C domain containing (JmjC) histone lysine demethylase (KDMs) (Xu et al., 2011; Chowdhury et al., 2011). This leads to increased methylation of histone (Kohli and Zhang, 2013) and O^6^-methylguanine-DNA methyltransferase (MGMT) promoter methylation (Turcan et al., 2012). Resulting epigenetic modification in the form of elevated H3K4 trimethylation (H3K4me3) (Gunn et al., 2023) at the telomerase reverse transcriptase (TERT) promoter facilitates transcription factors like Myc-Max to bind and activate TERT expression by promoting a more open chromatin structure, ultimately contributing to increased telomerase activity and cellular immortalization (Han et al., 2020). TERT is the catalytic subunit of telomerase and plays a pivotal role in telomere maintenance. Aberrant TERT methylation patterns are associated with inadequate treatment response, increased risk of recurrence and poor prognosis in various cancers. The mechanisms of gliomagenesis through hypermethylation are still being explored but likely involve inappropriate methylation silencing of tumor suppressor genes such as cyclin-dependent kinase inhibitor 2A/B (CDKN2A/B), retinoblastoma-associated protein (RB), RASSF1A (Wolter et al., 2001; Alonso et al., 2003; Kuo et al., 2013; Unruh et al., 2019) and aberrant activation of pro-malignant genes like epidermal growth factor receptor (EGFR), tissue factor (F3), prominin 1 (PROM1) (Unruh et al., 2019; de Botton et al., 2023; Losman and Kaelin, 2013; Bhavya et al., 2020; Unruh et al., 2019) and the oncogene platelet-derived growth factor receptor alpha (PDGFRA) (Flavahan et al., 2016). However, the epigenetic state is fluid and IDH mutant gliomas can lose much of their DNA methylation during tumor progression (Mazor et al., 2015; de Souza et al., 2018; Ceccarelli et al., 2016). Genetically similar stem-like cell populations have been identified in IDH-mutant gliomas (Venteicher et al., 2017; Tirosh et al., 2016) supporting the notion that epigenetic reprogramming induced by IDH mutations contributes to a differentiation block (Lu et al., 2012).

#### 2.3.2 Effects on tumor microenvironment

D-2-HG produced by IDH mutant glioma cells is actively exported into the tumor microenvironment (Linninger et al., 2018) where it suppresses antitumor T-cell function (Bunse et al., 2018), thereby promoting intratumoral immunosuppression (Bunse et al., 2018; Notarangelo et al., 2022; Mellinghoff et al., 2023). IDH-mutant gliomas exhibit reduced infiltration by tumor-infiltrating lymphocytes compared to their IDH wildtype counterparts. Epigenetic silencing of NKG2D ligands helps escaping surveillance by natural killer cells (Zhang et al., 2016). Also, 2-D-HG affects dendritic cell maturation and antigen presentation by innate lymphoid cells adding to reduced capacity to activate T cells (Friedrich et al., 2023). Other effects of elevated D-2-HG levels include promotion of angiogenesis via Vascular Endothelial Growth Factor Receptor VEGFR2 signaling, increased matrix metalloproteinase (MMP2) activity (Seok et al., 2019) and transformation of human astrocytes (Koivunen et al., 2012; Johannessen et al., 2016).

#### 2.3.3 Effect on normal cellular metabolic pathways

By interfering with normal cellular metabolic pathways, 2-D-HG depletes *α*-KG and NADPH from the Krebs cycle leading to disruption to redox balance (Badur et al., 2018) as evidenced by a ^13^C metabolic flux analysis (Grassian et al., 2014). Resulting accumulating oxidative damage is a hallmark of IDH mutant cancer biology (Han et al., 2020). The Krebs cycle is adjusted to compensate for these metabolic pathways and the loss of α-KG (Borodovsky et al., 2012) with the recruitment of several alternate non-Krebs-cycle carbohydrate sources (Maus and Peters, 2017; Ohka et al., 2014) including increased dependence on glutaminolysis for glutamate production (McBrayer et al., 2018; Seltzer et al., 2010). Glutamate dehydrogenase 2 is responsible for converting glutamate into α-KG and it is expressed at high levels in the brain. This enzyme mitigates the metabolic vulnerabilities associated with IDH mutations and IDH mutated glioma cells demonstrate increased sensitivity to glutaminase inhibition confirming the role of glutaminolysis as a key compensatory pathway in metabolic homoeostasis maintenance (Seltzer et al., 2010). However, this ‘glutamine addiction’ is not specific to IDH mutant gliomas and glutamine catabolism occurs a major metabolic change in many types of cancers (Lukey et al., 2013). This metabolic pathway is an attractive therapeutic target with various approaches being employed or under investigation to exploit the dependence of cancer cells on glutamine. These include depleting cancer cell glutamine supply (L-asparaginase in acute lymphoblastic leukemia), inhibiting cancer cell glutamine uptake (inhibition of the c-Myc-regulated transporter SLC1A5), use of glutamine mimetics (6-diazo-5-oxo-L-norleucine, acivicin, azaserine) and use of selective glutamines inhibitors (968 and BPTES) (Lukey et al., 2013). GLS inhibitor telaglenastat has shown promising results in preclinical studies and is currently being investigated in a clinical trial for patients with IDH mutant gliomas in combination with radiation therapy and temozolomide (NCT 2018). Due to its structural and functional similarity with glutamate, 2-D-HG contributes to seizures in patients with glioma (Reitman et al., 2011; Ruda et al., 2024).

IDH mutant glioma exhibit increased reliance on nicotinamide phosphoribotransferase (NAMPT) for nicotinamide adenine dinucleotide (NAD) biosynthesis (Tateishi et al., 2015). NAD depletion via concomitant NAMPT inhibition has been identified as a metabolic susceptibility of IDH1 mutant cancers and results in cytotoxicity triggered by autophagy (Tateishi et al., 2015). 2-D-HG also directly stimulates EgIN prolyl hydroxylase which in turn causes reduced HIF1α activity (Koivunen et al., 2012). HIF1α acts as a tumor suppressor in mouse models and its level are low in IDH mutant glioma (Blouw et al., 2003; Zagzag et al., 2000).

#### 2.3.4 Altered ribosome biogenesis and function

Ribosomal RNA (rRNA) 2’O-ribose methylation distinguishes IDH wildtype and mutant HGG. IDH wild-type glioblastomas display the most prominent defects in rRNA epitranscriptomics. The observed reduction in rRNA 2′-O-methylation (2′Ome) levels may be an indirect consequence of increased rRNA transcription linked to the heightened proliferative activity of glioblastoma cells. This upregulation in rRNA synthesis could potentially exceed the capacity of the rRNA 2′Ome modification machinery, leading to insufficient methylation. IDH mutant astrocytomas and oligodendrogliomas demonstrate elevated expression of ribosome biogenesis (RiBi) factors indicating that RiBi activity does not correlate directly with proliferative rate in HGG. These findings suggest that changes in rRNA epitranscriptomic modifications in HGG cannot be solely attributed to differences in ribosome biogenesis. IDH mutant glioma cells exhibit higher cytotoxicity to RNA polymerase I inhibitors, highlighting potential therapeutic implications (Paraqindes et al., 2023).

### 2.4 IDH mutations in non-glioma cancers and non-malignant conditions

In non-CNS malignancies, IDH mutations are identified in approximately 16% of acute myeloid leukemia (AML), myelodysplastic syndrome (DiNardo et al., 2015; Paschka et al., 2010; Mardis et al., 2009), 23% of intrahepatic cholangiocarcinoma (Borger et al., 2012), 56% of central/periosteal chondrosarcoma (Amary et al., 2011), colorectal carcinoma (Wood et al., 2007) and melanoma (Lopez et al., 2010; Shibata et al., 2011). The pathologic role of IDH1 mutations in these tumors have been supported by improvement in outcomes with the application of IDH1 mutation small molecule inhibitors such as olutasidenib and ivosidenib (Montesinos et al., 2022; Watts et al., 2023; de Botton et al., 2023). Patients with Ollier disease and Maffucci syndrome are at increased risk of developing IDH mutant gliomas in view of the underlying somatic IDH1/2 mutations (El Abiad et al., 2020; Pansuriya et al., 2011; Bonnet et al., 2016). These patients are typically younger and have multicentric lesions (Bonnet et al., 2016) and do not have 1p/19q codeletion.

## 3 Biology of IDH mutant HGG

### 3.1 Classification

**Figure 5:**
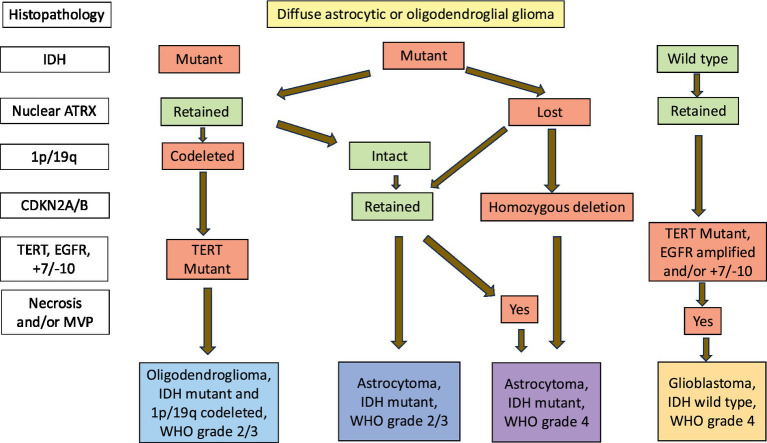
Integrated diagnostic classification algorithm for IDH mutant gliomas.

Historically, gliomas have been classified morphologically into four grades. Grades 1 and 2 tumours are typically slow-growing and well-differentiated and collectively termed as LGG. On the other hand, HGG are diffusely infiltrating, poorly differentiated and rapidly-growing tumors and are assigned grades 3 and 4 (Louis et al., 2001). In the last decade, there has been an unprecedent gain in the knowledge of molecular biology of CNS tumors which led to inclusion of the molecular characteristics into the diagnostic criteria in the latest edition of the WHO CNS Tumour Classification (Figure 5) (Louis et al., 2021).

IDH mutations are class-defining alterations in adult diffuse gliomas (Yan et al., 2009; Waitkus et al., 2016; Gupta et al., 2013) which are classified into 3 types: Astrocytoma, IDH mutant; Oligodendroglioma, IDH mutant and 1p/19 codeleted and Glioblastoma, IDH wildtype. Oligodendroglioma diagnosis requires loss of the short arm of chromosome 1 and the long arm of chromosome 19 (also known as 1p/19q codeletion) in addition to an IDH mutation. Astrocytomas have an IDH mutation without 1p/19q codeletion. Glioblastoma, IDH wildtype are diffuse and astrocytic gliomas in adults with microvascular proliferation or necrosis or TERT promoter mutation or EGFR gene amplification or +7/−10 chromosome copy number chnges.

### 3.2 Epidemiology

Although accounting for only 10% of childhood brain tumors, HGG including diffuse midline glioma (DMG) such as Diffuse Intrinsic Pontine Glioma (DIPG), are a leading cause of cancer-related death in children and adolescents (Rallis et al., 2022). Despite intensive multimodal therapy, prognosis for the 800–1,000 pediatric and AYA patients diagnosed each year in the United States (Ostrom et al., 2019) with these aggressive brain and/or spine tumors remain dismal, with 5-year overall survival (OS) < 10% (Fangusaro, 2012; Jones et al., 2017). Between 2017–2021, the incidence rate of IDH1/2 mutant astrocytoma was 0.46 per 100,000 population. IDH mutations were reported in 65, 54 and 3.6% of WHO grades 2, 3 and 4 astrocytomas, respectively, (Price et al., 2024). In pediatric population, the incidence rate of IDH1/2 mutant astrocytoma was 0.09 per 100,000 population (Ostrom et al., 2022) with 9.1% tumors histologically low grades (WHO grade 1 and 2) and 9% high grades (WHO grade 3 and 4) (Yeo et al., 2023).

### 3.3 IDH mutations in HGG

Although molecularly and clinically different, IDH mutant astrocytoma and oligodendroglioma share the same basic lineage. Single-cell RNA sequencing and experiments in genetically engineered mouse models reveal a common glial progenitor cell of origin with a similar developmental hierarchy consisting of proliferating neural stem-like cells and non-proliferating cells with astrocytic and oligodendroglial differentiation (Venteicher et al., 2017; Tirosh et al., 2016; Zong et al., 2015). Both IDH1 and IDH2 mutations are heterozygous and somatic in origin with prognostic and therapeutic significance (Ahrendsen et al., 2021). The missense mutations affect IDH1 codon 132 or IDH2 codon 172. With nearly 96% IDH mutant gliomas harboring an IDH1 mutation (Hartmann et al., 2009), the predominant alteration is IDH1:c.395G > A p. R132H (83–91%) (Balss et al., 2008), followed by IDH1:c.394C > T p. R132C (3.6–4.6%), p. R132G (0.8–2.5%), p. R132S (0.6–3.8%) and p. R132L (0.5–4.4%) of all IDH1 mutations (Balss et al., 2008). IDH2 mutations are much less frequent than IDH1with frequency of alterations in IDH2 mutations being 65% IDH2 p. R172K, 19% p. R172M and 16% p. R172W(Hartmann et al., 2009; Yan et al., 2009). For oligodendroglioma, approximately 90% of the IDH1 mutations are canonical (R132H), and there is a higher frequency of IDH2 mutations compared to IDH-mutant astrocytomas (Hartmann et al., 2009; Cancer Genome Atlas Research Network, 2015; Eckel-Passow et al., 2015).

### 3.4 Other co-existing genetic alterations in IDH mutant HGG

#### 3.4.1 IDH mutant astrocytoma

*TP53 and alpha thalassemia X-linked intellectual disability (ATRX):* Class-defining loss of function mutations in TP53 and ATRX genes are seen in approximately 90 and 70% of astrocytomas, respectively, (Cancer Genome Atlas Research Network, 2015; Jiao et al., 2012). ATRX mutations are associated with epigenomic dysregulation and telomerase dysfunction (Clynes et al., 2013) resulting in alternative lengthening of telomerase (Heaphy et al., 2011). Astrocytomas with Li-Fraumeni Syndrome/Germline TP53 mutations show remarkably selective occurrence of IDH1:c.394C > T p. R132C mutations (Watanabe et al., 2009).

*CDKN2A/B:* CDKN2A/B are tumor suppressor genes and their loss of expression contributes to malignant tumor progression by disrupting cell cycle regulation and enhancing cell proliferation (Sharpless, 2005). IDH mutant astrocytomas with CDKN2A/B homozygous deletions have significantly shorter survival (Yeo et al., 2023; Brat et al., 2020; Appay et al., 2019; Alentorn et al., 2015). Notably, the presence of CDKN2A/B loss denotes WHO Grade 4 disease independent of histopathological features (Louis et al., 2021). Similarly, hemizygous loss of CDKN2A/B is associated with reduced survival in IDH1/2 mutant astrocytomas (Ghosh et al., 2025). Both CDKN2A homozygous and hemizygous deletions are enriched in post-treatment, recurrent IDH mutant astrocytomas (Kocakavuk et al., 2023).

*Other genetic alterations:* Intrachromosomal and extrachromosomal focal amplifications drive tumor growth and are associated with negative prognosis in many cancers (Yi et al., 2022). Amplifications of CDK4/6, CCND2, PDGFRA, MYCN as well as RB1 mutation/homozygous deletion, MET alterations, PIK3R1/PIK3CA mutations, NOTCH1 mutations and TCF12 mutations are also associated with tumor progression & shorter survival in IDH mutant astrocytomas (Brat et al., 2020; Li et al., 2019; Broderick et al., 2004; Labreche et al., 2015; Kamoun et al., 2016; Aoki et al., 2018).

#### 3.4.2 IDH mutant oligodendroglioma

*TERT:* Oligodendrogliomas have loss of one copy of the entire short arm of chromosome 1 (1p) along with one copy of the long arm of chromosome 19 (19q) by definition and may harbor TERT activating promoter mutations (Miller et al., 2017; Koelsche et al., 2013; Lee et al., 2018) which are early and clonal events in gliomagenesis (Suzuki et al., 2015; Exner et al., 2019). TERT mutations present in the vast majority of oligodendroglioma and IDH wildtype glioblastoma are mutually exclusive with ATRX mutations and alternative lengthening of telomerase seen in astrocytoma (Cancer Genome Atlas Research Network, 2015; Eckel-Passow et al., 2015). TERT mutations lead to telomerase stabilization, cellular immortalization and proliferation (Hafezi and Perez Bercoff, 2020) and are characteristically absent in adolescents (Lee et al., 2018).

*CIC:* The CIC gene encoding the Protein capicua homolog is localized to chromosome 19q and the remaining allele is inactivated in 70% tumors (Eckel-Passow et al., 2015; Bettegowda et al., 2011). CIC represses receptor tyrosine kinase/MAPK pathway targets by its interaction with chromatin regulators (Wong and Yip, 2020) and its deletion promotes neural stem cell proliferation indicating a direct role in gliomagenesis (Yang et al., 2017).

*CDKN2A/B:* Similar to astrocytoma, CDKN2A/B homozygous deletion is an adverse prognostic factor in grade 3 oligodendroglioma (Fallon et al., 2004).

Other aberrations include FUBP1 somatic mutations in 20–30% (Sahm et al., 2012), combined CIC and FUBP1 mutations (Chan et al., 2014), NOTCH1 mutations in 15% and less frequent other NOTCH pathway mutations (Suzuki et al., 2015), SETD2, PIK3CA and SWI/SNF chromatin remodeling complex mutations (Cancer Genome Atlas Research Network, 2015). Patients with tumors harboring CIC or combined CIC and FUBP1 mutations as well as NOTCH1 mutations have shorter time to recurrence (Suzuki et al., 2015; Chan et al., 2014; Gleize et al., 2015). Other co-existing genetic alterations associated with tumor progression and shorter survival in oligodendroglioma include homozygous PIK3CA mutation (Broderick et al., 2004; Tateishi et al., 2019), TCF12 mutation (Labreche et al., 2015) and increased MYC signaling (Kamoun et al., 2016).

### 3.5 Clonal evolution and risk of malignant transformation of IDH mutant tumors

IDH mutant tumors molecularly evolve over time with IDH mutation being the initiating event with subsequent TP53 and ATRX alterations (astrocytomas) or 1p/19q codeletion (oligodendrogliomas) followed by TERT mutations (Bennett et al., 2025). Pediatric patients in the Toronto AYA cohort had IDH mutations alone or with only one of TP53 or ATRX alterations. Similarly, younger patients with oligodendroglioma had 1p/19q codeletion without TERT mutations. These patients had a median age of 22.2 years (range 8.9–35.1 years) compared to 32.7 years in those with TERT mutations and demonstrated improved survival (Bennett et al., 2025).

Malignant transformation in the form of histological and/or genomic progression from a LGG (WHO grade 2) to a HGG (WHO grades 3/4) at recurrence is known to occur in IDH mutant gliomas. The rate of malignant transformation is higher in YA [52 of 204 (25.5%)] compared to pediatric [6 of 48 (12.5%)] and older adults [18 of 127 (14.2%)]. YA have the shortest median time from diagnosis to malignant transformation (5.65 years) as opposed to older adults (8.82 years) and pediatric patients (11.19 years) (Lim-Fat et al., 2024).

### 3.6 IDH mutant HGG in children and AYA patients

Overall, IDH mutant tumors account for approximately 12% of all gliomas (Ostrom et al., 2019). The Centres for Disease Control and Prevention (CDC)‘s National Program of Cancer Registries and National Cancer Institute (NCI)‘s Surveillance, Epidemiology and End Results (SEER) Program started collecting data on IDH mutations and 1p/19q codeletion in 2018 (Miller et al., 2023). This information is included in the Central Brain Tumor Registry of the United States (CBTRUS) dataset. Over 40% of astrocytoma Grade 2–3, and around 2% of astrocytoma grade 4 are IDH mutant (Ostrom et al., 2019). Grade 3 oligodendrogliomas represent 0.4% of all brain tumors and among all oligodendroglial tumors, approximately one-third are classified as grade 3 (Ostrom et al., 2019). Patients with IDH mutant tumors are generally younger with a median age at diagnosis of 37 years for patients with grade 3 astrocytoma and 47 years for grade 4 astrocytoma and grade 3 oligodendroglioma (Hartmann et al., 2009; Eckel-Passow et al., 2015). In grade 3 astrocytoma and oligodendroglioma, there is a slight male predominance and M: F ratio is significantly lower in grade 4 astrocytoma compared to the wild type tumors (Cancer Genome Atlas Research Network, 2015; Ohgaki and Kleihues, 2013).

IDH-mutant gliomas exhibit a notable age-dependent prevalence. These tumors are rare in children under 9 years of age, accounting for only approximately 6% of pediatric tumors (Table 1). In contrast, their frequency in the AYA population is higher, ranging from 6 to 16.3% (Korshunov et al., 2015; Mackay et al., 2017). In the Toronto AYA cohort of 371 IDH mutant tumors, only 6 patients were younger than 15 years of age (Bennett et al., 2025). Due to their rarity in children, prevalence data and impact of IDH mutations on the prognosis comes from small cohort studies (Korshunov et al., 2019). Although most IDH mutant gliomas harbor IDH1 R132H mutation, frequency of non-canonical mutations, including IDH2, is higher in AYA population (Lim-Fat et al., 2024). Generally, younger age is considered a favorable prognostic factor in patients with IDH mutant gliomas; however, compared to pediatric and adult cohorts AYA have an inferior survival (Lim-Fat et al., 2024) as is the case for other cancer types.

**Table 1:** Pediatric/AYA studies with IDH mutant glioma.

Study/Publication (Reference)	Frequency of IDH mutant tumors	Outcome of IDH mutant tumors
Yeo et al. (2023)	78/851 (9.2%), low/high-grade glioma 52/570 (9.1%) LGG 25/277 (9%) HGG IDH1 68 (89.5%), IDH2 8 (10.5%) 2/378 (0.5%) 0–9 years old 76/473 (16.1%) 10–21 years old	High-grade astrocytoma 5-year PFS/OS 36.8%/84% 10-year PFS/OS 0%/22.2% High-grade oligodendroglioma 5-year PFS/OS 50%/50%
Pollack et al. (2011) COG ACNS0423	7/43 (16.3%) all patients with HGG IDH1 7 (100%), IDH2 0 (0%) 0/23 (0%) 0–13 years old 7/20 (35%) 14–21 years old	1-year EFS/OS 86%/100%
De Carli et al. (2009)	4/73 (5.5%), non-pilocytic glioma IDH1 4 (100%), IDH2 0 (0%) Median age 16 years	Not reported
Hartmann et al. (2009)	4/32 (12.5%), low/high-grade glioma IDH1 4 (100%), IDH2 0 (0%)	Not reported
Korshunov et al. (2015)	10/162 (6%), glioblastoma IDH1 10 (100%), IDH2 0 (0%) Age range 12–18 years, median age 15.5 years	5-year PFS 76%
Rodriguez et al. (2014)	1/12 (8.3%), grade 3 oligodendroglioma IDH1 1 (100%), IDH2 0 (0%) 18 years old	Not reported
Bennett et al. (2025)	371 of 633 (58%), IDH mutant Age range 8.9–39.8 years, median 31.7 years	Not reported
Lim-Fat et al. (2024)	8 of 379 (2.1%), grade ¾ Age range 8.9–77.17 years	Median OS Pediatric 12.12 years YA 18.8 years

### 3.7 Primary mismatch repair-deficient IDH mutant astrocytomas (PMMRDIA)

Primary mismatch repair-deficient IDH mutant astrocytomas (PMMRDIA) are HGG mainly found in children, adolescents, and young adults with a median age at diagnosis of 14 years (Dodgshun et al., 2020; Suwala et al., 2021). They are characterized by somatic IDH mutations along with germline mutations in mismatch repair genes (MLH1, PMS2, MSH6 and MSH2). These tumors are histologically high-grade IDH1 mutant astrocytomas and exhibit hypermutant genotype and microsatellite instability. PMMRDIA should be strongly suspected in AYA patients presenting with treatment-naïve IDH mutant gliomas with intact 1p/19q or loss of ATRX. These tumors show frequent inactivation of TP53, RB1 and activation of RTK/PI3K/AKT pathways with more than 60% presenting with unmethylated MGMT promoter. In contrast to IDH mutant tumors, PMMRDIA display hypomethylation within CpG islands (Dodgshun et al., 2020). Patients with PMMRDIA have the worst outcome of all IDH mutant tumors with a median survival of 15 months which is comparable to IDH wild-type glioblastoma (Table 2) (Suwala et al., 2021). The survival is similar for WHO grades 2–4 tumors unlike conventional IDH mutant astrocytoma. This poor survival is attributed to primary resistance of PMMRDIA toward alkylating agents like temozolomide (TMZ) mediated by MMR deficiency and high rate of unmethylated MGMT promoter. Although most PMMRDIA tumors are hypermutant, the tumour mutational burden (TMB) is not as high as patients with CMMRD or Lynch syndrome (Campbell et al., 2017). TMB for IDH mutant glioma is < 20 mutations/MB with the vast majority have TMB < 2 mutations/MB compared to PMMRDIA TMB of < 30 mutations/MB and MMR-deficient gliomas TMB between 80 and 380 mutations/MB (Wang et al., 2020). Consequently, these tumors may also be not as responsive to immune checkpoint inhibition as other MMR-deficient, hypermutant tumors in general are (Suwala et al., 2021). Based on their dismal outcome, PMMRDIA should be separated from other IDH mutant gliomas and considered as a distinct entity with therapeutic and prognostic implications (Touat et al., 2020).

**Table 2:** Pediatric/AYA studies with PMMRDIA.

Study/Publication (Reference)	PMMRDIA population	Outcome of IDH mutant tumors
Suwala et al. (2021)	32 with high-grade astrocytoma Age range 9–54 years Median age 14 years Inactivation of TP53 and RB1 Activation of RTK/PI3K/AKT pathway	Median OS 15 months
Yeo et al. (2023)	8 with MSH2/6 alterations out of 50 with IDH mutant HGG (16%)	Median OS 6.97 months
Dodgshun et al. (2020)	6 with IDH mutant tumours out of 50 with replication repair deficient HGG (12%)	Not reported
Bennett et al. (2025)	7 of 241 (3%) with IDH mutant astrocytoma Younger age at presentation, mean 21 years	

## 4 Clinical features and investigations

### 4.1 Symptoms

Focal or generalized seizures are the presenting symptom in majority of patients with seizures more commonly seen in IDH mutant glioma than glioblastoma (Schiff, 2015; Chen et al., 2017; Gonzalez Castro and Milligan, 2020). Seizures are secondary to the infiltrative nature of gliomas and structural similarity between 2-D-HG and glutamate activating neuronal NMDA receptors lowering seizure threshold (Chen et al., 2017). Seizures are more treatment refractory compared to IDH wildtype tumors (Jo et al., 2021).

### 4.2 Location

IDH mutations affect common glial precursor cells and are early events in gliomagenesis (Watanabe et al., 2009; Johnson et al., 2014). This precursor cell population is spatially and temporally restricted in the brain (Lai et al., 2011). Also, frontal lobes show elevated expression of glutamate dehydrogenase 2 (GDH2) which converts glutamate to *α*-KG. Mutant IDH enzyme utilize α-KG as a substrate which explains the predominant frontal lobe location of the IDH mutant tumors compared with IDH wildtype tumors (Cancer Genome Atlas Research Network, 2015; Chen et al., 2014; Waitkus et al., 2018). Less commonly oligodendrogliomas are seen in posterior fossa, basal ganglia and brainstem. Rarely, multifocal tumors and leptomeningeal dissemination at the time of initial diagnosis or relapse has been reported (Herrlinger et al., 2016).

### 4.3 Imaging

Magnetic resonance imaging (MRI) typically shows hyperintense signals in T2-weighted and T2-fluid attenuated inversion recovery (FLAIR) images. T2-FLAIR mismatch in the form of T2 hyperintensity with relative FLAIR hypointensity is a highly specific sign (specificity 100%, sensitivity 42%) of grade 3 astrocytoma (Park et al., 2021; Patel et al., 2017). Gadolinium enhancement is seen more commonly in grade 3 and grade 4 astrocytomas whereas rim enhancement surrounding central necrotic areas is seen in grade 4 astrocytoma. Marked contrast enhancement in grade 4 astrocytoma is associated with the presence of homozygous CDKN2A deletion, although with low sensitivity (80%) and specificity (58%) (Park et al., 2023). In grade 3 oligodendroglioma, gadolinium enhancement is seen in >70% of tumors (Roux et al., 2020; Suchorska et al., 2019; Khalid et al., 2012) and is associated with microvascular proliferation and less favorable prognosis (Halani et al., 2018). Oligodendrogliomas show higher microvascularity and higher vascular heterogeneity than astrocytomas (Latysheva et al., 2019). They involve the cortex more frequently and calcification identified on computed tomography (CT) or susceptibility-weighted imaging (SWI) is highly specific for oligodendrogliomas. Magnetic resonance spectroscopy (MRS) can non-invasively detect IDH mutant glioma by demonstrating elevated 2-D-HG levels (Branzoli et al., 2018) as it can accumulate to very high (1–50 mM) concentration in the tumor (Zhou et al., 2018; Andronesi et al., 2012). MRS maps actual areas of involvement beyond the increased T2 signal supporting the infiltrative nature of these gliomas. D-2-HG signaling by MRS is also being evaluated for response assessment (Choi et al., 2016; Andronesi et al., 2016; de la Fuente et al., 2016). Response to therapy is assessed by RANO (Wen et al., 2010; Wen et al., 2017) and RANO 2.0 criteria (Ellingson et al., 2022) and some pediatric and AYA trials have incorporated RAPNO criteria. Molecular positron emission tomography (PET) imaging using amino acid tracers may be promising for the evaluation of IDH mutant gliomas, especially in the setting of treatment with IDH inhibitors (Albert et al., 2024). Recently, the use of generative artificial intelligence (AI)-based augmentation (GAA) with realistic and diverse imaging was shown to outperform neuroradiologists in predicting IDH status in gliomas (Moon et al., 2024).

## 5 Pathology

### 5.1 Proliferation, growth pattern and vasculature

The large French national POLA cohort findings defined 3 groups of CNS WHO grade 3 oligodendrogliomas based on mitotic index, microvascular proliferation (MVP) and necrosis. Group 1 tumors have high mitotic count but the absence of MVP, Group 2 with presence of MVP and absence of necrosis and Group 3 with presence of both MVP and necrosis (Figarella-Branger et al., 2014).

### 5.2 Immunophenotype

Oligodendrogliomas retain nuclear expression of ATRX (Liu et al., 2012; Reuss et al., 2015) and lack widespread nuclear p53 staining differentiating them from astrocytomas (Cancer Genome Atlas Research Network, 2015; Suzuki et al., 2015). Loss of nuclear ATRX is enough to establish the diagnosis of astrocytoma without additional testing for 1p/19q codeletion (Louis et al., 2018).

### 5.3 DNA methylation profiling

#### 5.3.1 IDH mutant vs. wild type gliomas

The DNA methylation profiling clearly distinguishes IDH mutant gliomas from their wild type counterparts by the glioma CpG island methylator phenotype (G-CIMP) (Turcan et al., 2012; Wiestler et al., 2014; Noushmehr et al., 2010). G-CIMP-high (IDH mutant) and G-CIMP-low (IDH wild type) signatures are determined based on the extent of hypermethylation and the latter confers a worse prognosis (Ceccarelli et al., 2016).

#### 5.3.2 IDH mutant glioma subclasses

DNA methylation profiling is a method used to differentiate cancer cells from normal tissue. Consistent with the finding of a shared histogenesis and developmental hierarchy, DNA methylation also shows high similarity between three groups compared to IDH wildtype glioma and IDH mutant non-glioma tumors (Venteicher et al., 2017). The current version of the DNA methylation-based CNS tumor classification system separates IDH mutant gliomas into three groups per 2021 WHO CNS tumor classification (Capper et al., 2018).

- *Subclass astrocytoma-A IDH*: This class accounts for all grade 2 and some grade 3 astrocytoma. This subclass does not have characteristic copy number variation (CNV) pattern.
- *Subclass high-grade astrocytoma-A IDH, HG*: This class accounts for some grade 3 and most of the grade 4 astrocytoma. CNV pattern shows similarities with subclass-A IDH but has a higher number of total and complex changes and frequent loss on chromosome 9p including the CDKN2A/B locus.
- *Subclass 1p/19q-codeleted oligodendroglioma-O IDH*: includes both WHO Grade 2 and 3 tumors. These tumors are characterized by hypermethylation and concurrent downregulation of TP73 antisense RNA 1 (TP73-AS1) gene (Sturm et al., 2012). CNV of this group exhibits 1p/19q codeletion in all cases and loss of chromosome 4p/q in most of the tumors.

These three groups are closely related and classed under the “IDH glioma” methylation class family. Due to the strong association between the DNA methylation class and the presence of an IDH mutation, the methylation class is considered a proof of IDH mutational status (Capper et al., 2018) Methylation classes A IDH, A IDH HG and O IDH show a high overlap with methylation groups LGm1, LGm2 and LGm3, respectively, as described in The Cancer Genome Atlas (TCGA) dataset of Ceccarelli et al. in 2016 (Ceccarelli et al., 2016). The distinction between subclass-A IDH and subclass-A IDH HG has prognostic implications as confirmed in independent datasets (Kling et al., 2024; Tesileanu et al., 2021).

#### 5.3.3 IDH mutant infratentorial glioma

Infratentorial astrocytoma form a distinct methylation cluster and a discrete subgroup within IDH-mutant astrocytoma (Banan et al., 2020). These infratentorial tumors have high frequency of non-canonical IDH mutations thereby failing to be detected by immunohistochemistry (IHC). Incidence of ATRX mutations is also much lower in these tumors. Patients with infratentorial IDH mutant astrocytomas have significantly better clinical outcomes compared to those with diffuse midline gliomas harboring the H3K27M mutation, but significantly worse outcomes than patients with supratentorial IDH mutant astrocytomas (Banan et al., 2020).

#### 5.3.4 DNA methylation versus WHO tumor grading

A recent analysis of 98 tumors revealed that the current WHO grading criteria for IDH mutant astrocytomas had limited prognostic utility. In contrast, stratification based on DNA methylation profiles provided a more accurate prediction of overall survival. CNS WHO grade 3 patients were separated into low- and high-grade IDH-mutant astrocytoma groups based on DNA methylation profiling, eliminating the necessity for an intermediate histologic grade (Galbraith et al., 2024). On the other hand, Deep learning from histoPathoLOgy and methYlation (DEPLOY), a deep learning model classifying CNS tumors, was trained to predict tumor types including IDH mutant gliomas from hematoxylin and eosin (H&E) sections, (Hoang et al., 2024). DEPLOY comprises three distinct computational modules: (1) a “direct model” that classifies CNS tumors directly from histopathology slides; (2) an “indirect model” that first predicts DNA methylation beta values from slide images, which are then used to infer tumor classification; and (3) a “demographic model” that predicts tumor types directly from routinely collected patient demographic data. DEPLOY accurately predicted DNA methylation beta values from histopathological images. Utilizing a ten-class classification model trained on an internal cohort of 1,796 patients, DEPLOY predicted tumor categories in three independent external test datasets comprising a total of 2,156 patients. On high-confidence predictions, the model achieves an overall accuracy of 95% and a balanced accuracy of 91%. These findings highlight the potential of DEPLOY as a clinical decision-support tool to assist pathologists in the rapid and accurate diagnosis of CNS tumors potentially obsoleting the need for molecular studies.

### 5.4 MGMT promoter methylation

MGMT promoter is methylated in 85 and 98% of IDH mutant astrocytomas and oligodendrogliomas, respectively, (Turcan et al., 2012; Capper et al., 2018; Mollemann et al., 2005; Mulholland et al., 2012; Horbinski et al., 2021) and this phenotype is part of the G-CIMP signature (Noushmehr et al., 2010; van den Bent et al., 2011; Laffaire et al., 2011). MGMT promoter methylation in IDH mutant gliomas is a result of metabolic alterations including increased production of 2-D-HG and genome-wide methylation (Turcan et al., 2012; Lu et al., 2012; Figueroa et al., 2010). Unlike IDH wildtype glioblastoma, there is no clear response prediction with MGMT promoter methylation (Wick et al., 2013) but it is associated with improved survival in IDH mutant gliomas (Lam et al., 2022).

### 5.5 Machine-learning platforms

A computational pipeline, APOLLO (rAman-based PathOLogy of maLignant gliOma) using spontaneous Raman spectroscopy, was employed to obtain molecular fingerprints from formalin-fixed paraffin-embedded (FFPE) tissue samples of 46 patients with known DNA methylation subtypes. APOLLO distinguished tumor from non-tumor tissue and IDH1 wildtype from IDH1 mutant. It identified IDH mutant gliomas to be highly abundant with cholesterol ester levels. APOLLO also demonstrated strong discriminative capability in resolving clinically relevant glioma methylation subtypes, specifically distinguishing between the G-CIMP-high and G-CIMP-low molecular subtypes within IDH1-mutant gliomas. Overall, this workflow has a potential to uncover biologically relevant information from FFPE slides, paving the way for its application to archived tissues (Lita et al., 2024).

## 6 Diagnosis

The diagnosis of IDH-mutant gliomas is based on histopathological and molecular features. Among glioma subtypes, IDH1/2 mutant glioma has undergone one of the most significant reclassifications in the shift from histopathologic to molecular criteria, with nearly half of these tumors formerly diagnosed as glioblastoma or other non-astrocytic gliomas (Brat et al., 2020). Immunohistochemistry for IDH1 R132H or IDH2 R172 mutations is a widely used diagnostic tool. Additional molecular markers, such as 1p/19q co-deletion and ATRX mutations, can aid in the diagnosis of oligodendrogliomas. Unbalanced translocation between the chromosomes 1 and 19 eliminates the 1p/19q fusion chromosome leaving only one copy of 1p and 19q each (Jenkins et al., 2006).

### 6.1 WHO diagnostic criteria for IDH mutant astrocytoma

**Table:** 

*Essential* A diffusely infiltrating glioma *AND* IDH1 codon 132 or IDH2 codon 172 missense mutation *AND* Loss of nuclear ATRX expression or ATRX mutation *OR* Exclusion of combined whole-arm deletions of 1p and 19q
*Desirable* TP53 mutation or strong nuclear expression of p53 in > 10% of tumor cells Methylation profile of astrocytoma, IDH mutant Astrocyte differentiation by morphology Louis et al. (2021).

### 6.2 WHO diagnostic criteria for oligodendroglioma, IDH-mutant and 1p/19q codeleted

**Table:** 

*Essential* A diffusely infiltrating glioma *AND* IDH1 codon 132 or IDH2 codon 172 missense mutation *AND* Combined whole-arm deletions of 1p and 19q
*Desirable* DNA methylome profile of oligodendroglioma, IDH mutant and 1p/19q codeleted Retained nuclear expression of ATRX TERT promoter mutation Louis et al. (2021).

### 6.3 Diagnostic tests for the diagnosis of IDH mutant HGG

Several methods can be deployed for determining the status of molecular markers and achieving an integrated histomolecular diagnosis of IDH mutant glioma as described in Table 3.

**Table 3:** Diagnostic tests for confirmation of IDH mutant glioma.

Pathological marker	Diagnostic modality	Diagnostic/Prognostic role
IDH1/2 mutation	Immunohistochemistry Gene sequencing	IDH1/IDH2 immunopositivity or mutation differentiates IDH mutant gliomas from IDH wild-type gliomas (Bell et al., 2020)
1p/19q codeletion	Fluorescent *In Situ* Hybridization (FISH)	Whole-arm chromosomal losses of chromosomes 1p and 19q differentiate IDH mutant oligodendroglioma from IDH mutant astrocytoma (Brown et al., 2021; Woehrer and Hainfellner, 2015) 1q and 19p co-polysomy concurrent with 1p/19q codeletion associated with earlier recurrence and shorter survival in IDH mutant oligodendroglioma (Chen et al., 2019; Snuderl et al., 2009)
ATRX mutation	Immunohistochemistry Gene sequencing	Loss of nuclear ATRX immunoreactivity or identification of ATRX mutation differentiates IDH mutant astrocytoma from IDH mutant oligodendroglioma (Ikemura et al., 2016)
TP53 mutation	Immunohistochemistry Gene sequencing	Strong and diffuse immunopositivity or identification of TP53 mutation differentiates IDH mutant astrocytoma from IDH mutant oligodendroglioma (Takami et al., 2015)
Proliferation: Ki67, MCM6, Mitotic activity Anaplasia Necrosis Microvascular proliferation	Histopathology	Differentiate between Grades 2–4 IDH mutant astrocytoma and Grades 2–3 IDH mutant oligodendroglioma (Brat et al., 2020)
CDKN2A and CDKN2B deletion	Gene sequencing Copy number profile from DNA methylation	Homozygous deletion of CDKN2A and/or CDKN2B associated with poor prognosis and IDH mutant astrocytoma; marker of WHO Grade 4 disease (Korshunov et al., 2019; Shirahata et al., 2018) Allelic losses of 9p21.3 (CDKN2A gene locus) and homozygous deletion of CDKN2A associated with poor survival in IDH mutant Grade 3 oligodendroglioma; marker of WHO Grade 3 disease (occurs in < 10% patients)
TERT promoter mutation	Gene sequencing	TERT promoter mutations seen in > 95% patients with IDH mutant oligodendroglioma and uncommonly in IDH mutant astrocytoma (Koelsche et al., 2013; Suzuki et al., 2015; Arita et al., 2013; Labussiere et al., 2014); TERT wild-type tumors seen in teenage patient (Lee et al., 2018)

### 6.4 Dual-genotype IDH mutant gliomas

Although most of the IDH mutant gliomas have defining molecular alterations differentiating between astrocytoma and oligodendroglioma, these are not mutually exclusive. There are rare cases displaying distinct regions of oligodendroglioma morphology and 1p/19q codeletion while other regions show astrocytic morphology, ATRX loss and TP53 mutations (Barresi et al., 2017; Huse et al., 2015; Qu et al., 2007). The latest WHO system does not have precise classification for these tumors but a layered diagnostic approach with morphological findings, molecular alterations and an “NEC” designation is suggested (Louis et al., 2018).

## 7 Current and investigational treatment strategies for IDH mutant HGG

**Figure 6:**
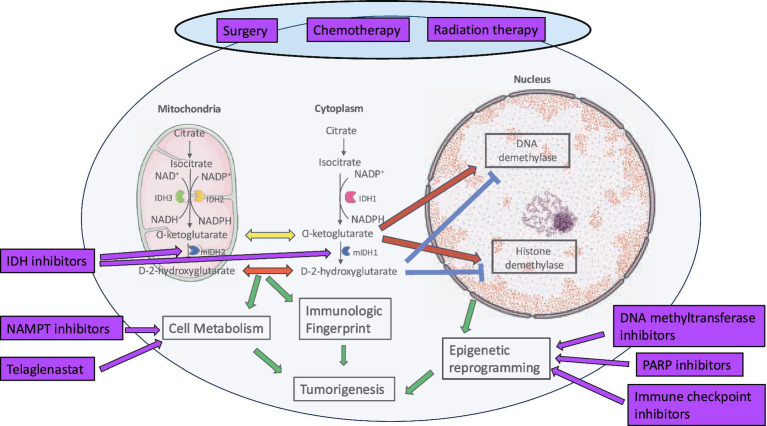
Current and investigational therapies for IDH mutant HGG.

IDH mutation is an independent favorable prognostic marker in adult glioma. However, this is only true for treated tumors and may be associated with increased sensitivity of these tumors to chemotherapy and RT (Yan et al., 2009; Houillier et al., 2010; Sanson et al., 2009). Figure 6 provides an overview of the current and experimental strategies for the treatment of IDH mutant glioma.

*Surgery:* Gross total resection (GTR) is associated with better prognosis across gliomas of all grades and subtypes (Molinaro et al., 2020; Grabowski et al., 2014) and the extent of resection has been confirmed to be a prognostic factor in IDH mutant oligodendroglioma (Wick et al., 2009). OS is better when patients undergo maximal surgical resection of the T2/FLAIR hyperintense areas (Karschnia et al., 2021; Smith et al., 2008; Beiko et al., 2014) with some evidence for supramaximal resection improving PFS (Motomura et al., 2021; Rossi et al., 2021). In 15 paediatric patients with IDH mutant HGG, there was a trend toward better PFS with better extent of resection at the time of initial diagnosis. 5-year PFS was 66.4% (median PFS 7.5 years) for patients with GTR compared to 19.9% (median PFS 4 years) for patients with subtotal resection (STR) or biopsy (Yeo et al., 2023). With the evidence of better survival, supramaximal safe resection or FLAIRectomy is recommended for IDH mutant HGG. This involves removal of all contrast-enhancing disease and non-contrast-enhancing T2/FLAIR areas (Young et al., 2023).

*Adjuvant therapies:* There is a definite survival advantage with adjuvant chemoradiotherapy. The NOA-04 clinical trial compared primary monochemotherapy to primary RT for patients with newly diagnosed anaplastic gliomas. The PFS and OS with RT alone were similar to procarbazine, lomustine and vincristine (PCV) or TMZ alone for all patients including those with IDH mutant gliomas (Wick et al., 2016). The results of the NOA-04 and 3 other randomized clinical trials do not support the use of primary monochemotherapy. The recommended RT dose for Grade 3 and 4 gliomas is 59.4 Gy or 60 Gy administered in 1.8 Gy or 2.0 Gy fractions, respectively, (Nabors et al., 2020). Post-RT pseudoprogression tends to occur later than in IDH wildtype glioblastomas and is not only common but also often misdiagnosed. During the first two years following radiotherapy, the potential for pseudoprogression should be carefully evaluated (Seyve et al., 2023). A phase 2 clinical trial, NRG-BN005, is randomizing grade 2 and 3 IDH mutant glioma patients to proton or photon RT to evaluate the role of these modalities on cognition (NCT, 2017).

### 7.1 Standard of care

The European Association of Neuro-Oncology (EANO) (Weller et al., 2021), the American Society of Clinical Oncology (ASCO)(Mohile et al., 2022), and the Society for Neuro-Oncology (SNO) (Miller et al., 2023) have developed guidelines for the treatment of diffuse gliomas of adulthood, including IDH mutant gliomas.

#### 7.1.1 IDH mutant grade 3 astrocytoma

The current standard of care for patients with IDH mutant Grade 3 astrocytoma is maximal safe surgical resection followed by involved field RT and maintenance TMZ (Mohile et al., 2022; Weller et al., 2021; Wick et al., 2009; Wick et al., 2016). CATNON (EORTC study 26,053–22,054) results showed survival benefit from adjuvant TMZ in adults with IDH mutant anaplastic astrocytoma (van den Bent et al., 2017; van den Bent et al., 2021). Korean Society of Neuro-Oncology Group study KNOG-1101 showed improved survival in adults with non codeleted anaplastic gliomas treated with concurrent and adjuvant TMZ with RT compared to only RT. However, there was no survival difference in a subgroup analysis of patients with IDH1 mutation which was likely due to the small sample size (Hwang et al., 2020).

#### 7.1.2 IDH mutant grade 3 oligodendroglioma 1p/19q codeleted

The current standard of care for patients with IDH mutant Grade 3 oligodendroglioma is maximal safe surgical resection followed by involved field RT and maintenance PCV polychemotherapy (Mohile et al., 2022; Weller et al., 2021; Cairncross et al., 2013; van den Bent et al., 2013). EORTC Brain Tumour Group Study 26,951 was a randomized trial comparing efficacy of RT alone and RT followed by six cycles of PCV chemotherapy in adults with anaplastic oligodendroglioma. Adjuvant PCV was more beneficial in patients with 1p/19q codeleted tumors and IDH mutation was of prognostic significance as well (van den Bent et al., 2013). Radiation Therapy Oncology Group (RTOG) Study 9,402 compared intensive PCV chemotherapy before RT to RT alone. Chemoradiotherapy was shown to be a highly effective therapy with survival benefit over RT alone in patients with 1p/19q codeleted oligodendroglioma (Cairncross et al., 2013). There are ongoing phase 3 clinical trials trying to identify superior postsurgical treatment options and improve brain function and quality of life in patients with grade 2/3 oligodendroglioma (NCT, 2009, 2015; Wick et al., 2022).

#### 7.1.3 IDH mutant grade 4 astrocytoma

The current standard of care for patients with IDH mutant WHO Grade 4 astrocytoma is either RT + adjuvant TMZ (similar to treatment for IDH mutant Grade 3 astrocytoma) or concurrent and adjuvant TMZ with RT (similar to treatment for IDH wildtype glioblastoma WHO Grade 4) (Mohile et al., 2022; Weller et al., 2021).

#### 7.1.4 TMZ associated hypermutant phenotype

Therapy with the alkylating chemotherapy drug TMZ leads to acquired defects in DNA mismatch repair genes and a hypermutant phenotype in approximately 60% of oligodendrogliomas and 30% of astrocytomas (Eckel-Passow et al., 2015; Johnson et al., 2014; Bell et al., 2015). MGMT promoter methylated gliomas are more commonly associated with TMZ-induced hypermutation (Mathur et al., 2020). These tumors are more aggressive and resistant to further therapy with alkylator agents on account of mismatch repair (MMR) pathway mutations (Touat et al., 2020; Leelatian et al., 2021). However, the benefit of TMZ therapy with RT outweigh the risks and leads to improved survival compared to RT alone (van den Bent et al., 2021).

### 7.2 Clinical trials of chemoradiotherapy enrolling patients with IDH mutant glioma

Various clinical trials (Table 4) have investigated the role of adjuvant chemoradiotherapy in patients with IDH mutant HGG.

**Table 4:** Clinical trials with chemoradiotherapy for patients with IDH mutant HGG.

Clinical trial (Ref)/ Phase	Patient population	Intervention (IDH mutant glioma patients)	Outcome
NOA-04 (216, 223) Phase 3	Newly diagnosed anaplastic glioma Age ≥ 18 years	Randomization between PCV or TMZ monotherapy (Borodovsky et al., 2012) vs. RT (Badur et al., 2018)	Similar survival between RT vs. chemotherapy including those with IDH mutant glioma
CATNON (EORTC study 26,053–22,054) (van den Bent et al., 2017; van den Bent et al., 2021) Phase 3	Newly diagnosed anaplastic glioma without 1p/19q codeletion Age ≥ 18 years	Randomization between RT alone (Clynes et al., 2013) vs. RT + concurrent TMZ (Brat et al., 2020) vs. RT + adjuvant TMZ (Alentorn et al., 2015) vs. RT + concurrent and adjuvant TMZ (Alentorn et al., 2015)	Compared to RT alone patients with IDH mutant glioma receiving any TMZ had better PFS (77 vs. 34.2 months) and OS (114.4 vs. 68.2 months) Survival benefit only from adjuvant TMZ
KNOG-1101 (Hwang et al., 2020) Phase 2	Newly diagnosed anaplastic glioma without 1p/19q codeletion Age ≥ 18 years	Randomization between RT alone (Parsons et al., 2008) vs. RT + concurrent and adjuvant TMZ (Mardis et al., 2009)	Similar survival between RT vs. RT with chemotherapy
EORTC 26951 (van den Bent et al., 2013) Phase 3	Newly diagnosed anaplastic oligodendroglioma Age ≥ 18 years	Randomization between RT alone (Pirozzi and Yan, 2021) vs. RT + PCV Chemotherapy (Turcan et al., 2012)	*1p/19q codeleted anaplastic glioma* Significantly longer OS/PFS in the PCV/RT arm (not reached/157 months) vs. RT only arm (112 months/50 months) *IDH mutated anaplastic glioma* Significantly longer OS/PFS in the RT/PCV arm (not reached/71 months) vs. RT only arm (65 months/36 months)
RTOG 9402 (Cairncross et al., 2013) Phase 3	Newly diagnosed anaplastic oligodendroglioma Age ≥ 18 years	Randomization between RT alone (Friedrich et al., 2023) vs. PCV chemotherapy + RT (Venteicher et al., 2017)	*1p/19q codeleted anaplastic glioma* Significantly longer OS/PFS in the PCV/RT arm (14.7 years/8.4 years) vs. RT only arm (7.3 years/2.9 years)
CODEL (NCT00887146) (NCT, 2009) Phase 3	Newly diagnosed codeleted 1p/19q anaplastic glioma Age ≥ 18 years	Randomization between RT with adjuvant PCV vs. RT with concurrent and adjuvant TMZ	Ongoing
NCT03528642 (NCT, 2018) Phase 1b	Newly diagnosed IDH mutant glioma Age ≥ 16 years	GLS inhibitor telaglenastat with RT and TMZ	Ongoing
POLCA (NCT, 2015) Phase 3	Newly diagnosed codeleted 1p/19q anaplastic glioma Age ≥ 18 years	Randomization between RT with adjuvant PCV vs. PCV alone	Ongoing
NOA-18/IMPROVE CODEL (Wick et al., 2022) Phase 3	Newly diagnosed codeleted 1p/19q grade 2/3 oligodendroglioma Age ≥ 18 years	Randomization between RT with adjuvant PCV vs. lomustine and TMZ (CETEG)	Ongoing

### 7.3 Preclinical studies of IDH inhibitors and other targeted therapies

IDH mutations provide a biologically rationale drug target. However, owing partly to methylation-induced suppression of protooncogenes, most patient-derived IDH mutant gliomas do not grow well in culture (Piaskowski et al., 2011) with relatively few preclinical studies published. These studies confirm antitumor activity of IDH inhibitors either alone (Table 5) or in combination with radiation therapy (RT), chemotherapy and immunotherapy. Similarly, PARP inhibitors alone or with TMZ have been shown to be effective against a variety of IDH mutant tumors (Gbyli et al., 2022; Sulkowski et al., 2017; Lu et al., 2017; Pusch et al., 2017).

**Table 5:** Preclinical studies of IDH inhibitors.

Treatment	Type of IDH inhibitor	Cell line	Animal model	Results
AGI-5198 (Rohle et al., 2013)	1^st^ generation IDH1 R132H specific inhibitor	TS603 IDH mutant grade 3 oligodendroglioma	Mouse xenografts	Reversed histone H3K9me3 methylation Slowed tumor growth
Vorasidenib (AG-881) (Konteatis et al., 2020)	Dual IDH1/2 inhibitor	TS603 IDH1 R132H glioma-sphere U87MG pLVX IDH2 R140Q engineered	Mouse, rat and monkey orthotopic xenografts	Brain penetrant > 97% reduction in 2-D-HG production
Vorasidenib Bay-1436032 (Radoul et al., 2021)	Dual IDH1/2 inhibitors	BT257 IDH mutant astrocytoma SF10417 IDH1 mutant oligodendroglioma U87IDHmut IDH1 R132H engineered	Orthotopic mouse xenografts	Reduced 2-D-HG production Increased glutamate, the sum of glutamate and glutamine and N-acetylaspartate Survival benefit
Bay-1436032 (Pusch et al., 2017)	IDH1/2 inhibitor	Glioblastoma cell line LN229 Human embryonic kidney cell line HEK293 Sarcoma cell line 1,080	Orthotopic mouse model with NCH551b cells	Reduce 2-D-HG levels Survival benefit
DS-1001b (Machida et al., 2020)	IDH1 inhibitor	GBM	A1074 Orthotopic mouse model	Reduction of 2-D-HG Good BBB penetration Survival benefit
Vorasidenib + neopeptide vaccines Vorasidenib + anti-PD1-antibody (Chuntova et al., 2022)	2^nd^ generation dual IDH1/2 inhibitor	dTG-IDH^R132H^	Double transgenic mouse xenografts	Reduction of 2-D-HG Survival benefit
AGI-5198 + RT + TMZ AGI-5198 + RT + TMZ + anti-PD1-antibody (Kadiyala et al., 2021)	1^st^ generation IDH1 R132H specific inhibitor	Mouse IDH1 mutant-OVA Human SJGBM2-IDH1 mutant Human SF10602	Genetically engineered mouse model	Survival benefit Reduced T cell exhaustion, generation of memory CD8 + T cells leading to immunological memory
AGI-5198 + RT	1^st^ generation IDH1 R132H specific inhibitor	Human SF10602 Human MGG119 Human LC1035	Genetically engineered mouse model	Decreased expression of DNA damage response regulator ZMYND8 Survival benefit

### 7.4 IDH1/2 inhibitors clinical trials of targeted therapies for patients with IDH mutant glioma

A number of clinical trials with targeted therapies are being conducted for patients with IDH mutant gliomas (Table 6).

**Table 6:** Targeted therapies for IDH mutant gliomas.

Clinical trial (Ref)	Patient population	Intervention (IDH mutant glioma patients)	Outcome
INDIGO Phase 3	IDH mutant gliomas grade 2 Age ≥ 12 years	Vorasidenib 40 mg once a day (168 of 331)	Improved PFS and time to second intervention
Ivosidenib in advanced solid tumors (Mellinghoff et al., 2020) Phase 1	Advanced IDH1 mutant glioma Age ≥ 18 years	Ivosidenib dose escalation and expansion (30 out of 66 grade 3/4)	Ivosidenib 500 mg/d had a favorable safety profile, prolonged disease control and reduced growth of nonenhancing tumors
Vorasidenib in advanced solid tumors (Mellinghoff et al., 2021) Phase 1	Advanced IDH1/2 mutant glioma Age ≥ 18 years	Vorasidenib dose escalation and expansion (26 out of 52 grade 3/4)	Ivosidenib 50 mg/d had a favorable safety profile, prolonged disease control and reduced growth of nonenhancing tumors
NCT03343197 (Mellinghoff et al., 2023) Phase 1	Recurrent nonenhancing IDH1 mutant glioma Age ≥ 18 years	Randomized trial of vorasidenib and ivosidenib (6 patients with grade 3)	Good tolerability for both agents Tumor-to-plasma concentration higher for vorasidenib
NCT03684811 (NCT, 2018) Phase 1/2	Relapsed/refractory IDH1 mutant glioma Age ≥ 18 years	Olutasidenib (FT-2102) 150 mg BD	Well tolerated with preliminary evidence of clinical activity
Bay1436032 (Wick et al., 2021) Phase 1	Advanced IDH1 R132 mutant glioma Age ≥ 18 years	Bay1436032 dose escalation and expansion (55 grade 2–4)	Well tolerated and activity in a small subset of grade 2–3 patients
DS1001-A-J101 (Natsume et al., 2023) Phase 1	Recurrent/progressive IDH1 R132 mutant glioma Age ≥ 18 years	DS-1001 (safusidenib) dose escalation (30 grade 3–4)	Safe and active
I9Y-OX-JDHC (NCT, 2020) Phase 1	Advanced IDH1/2 mutant glioma Age ≥ 18 years	LY3410738 dose escalation and expansion	Ongoing
NCT04762602 (NCT, 2021) Phase 1	Advanced IDH1/2 mutant solid tumors Age ≥ 18 years	HMPL-306 dose escalation and expansion	Ongoing
NOA-16 Platten et al., 2021 Phase 1	Newly diagnosed IDH1 R132H mutant grade 3/4 astrocytoma Age ≥ 18 years	IDH1 R132H long peptide vaccine (IDH-1 vac) post RT alone or with TMZ (Hayes et al., 2020)	IDH-1 vac safe to administer and produced T helper immune response
AMPLIFY-NEOVAC (Bunse et al., 2022) Phase 1	Recurrent IDH1 R132H mutant glioma Age ≥ 18 years	Randomized 3-arm trial comparing ICI avelumab with or without IDH1-vac	Completed, results pending
NCT03666559 (NCT, 2018) Phase 2	Recurrent IDH mutant glioma Age ≥ 18 years	Azacitidine in grade 2/3 gliomas	Ongoing
NCT03922555 (NCT, 2019a) Phase 1	Recurrent IDH mutant glioma Age ≥ 18 years	ASTX727 in nonenhancing gliomas	Ongoing
OLAGLI (Ducray et al., 2021) Phase 2	Recurrent IDH mutant glioma Age ≥ 18 years	Olaparib (Yan et al., 2009)	Well tolerated 5% partial response 37% stable disease
COG ACNS1731 (Karajannis et al., 2025) Stratum 2 Phase 2	Newly diagnosed IDH mutant HGG Age ≥ 3 to ≤ 25 years	RT, veliparib, TMZ (Parsons et al., 2008)	2-year EFS 43% 2-year OS 86%
NCT05076513 (NCT, 2021) Phase 0	Recurrent IDH1 mutant grade 2–4 astrocytoma Age ≥ 18 years	Arm B Niraparib monotherapy	Ongoing
NCT03914742 (NCT, 2019c) Phase 1/2	Recurrent IDH mutant grade 2–3 astrocytoma Age ≥ 18 years	BGB290 + TMZ	Ongoing
NCT03991832 (NCT, 2019b) Phase 2	Recurrent IDH mutant glioma Age ≥ 18 years	Arm A Olaparib + Durvalumab	Ongoing
Phase 1b trial	Newly diagnosed IDH mutant glioma Age ≥ 16 years	GLS inhibitor telaglenastat with RT and TMZ	Ongoing

#### 7.4.1 IDH1/2 inhibitors

Phase 1 trials of ivosidenib and vorasidenib confirmed safety and activity in patients with nonenhancing IDH mutant glioma (Mellinghoff et al., 2020; Mellinghoff et al., 2021). These early trials showed efficacy of IDH inhibitors in nonenhancing tumors regardless of grade suggesting role of additional molecular alteration in advanced stages of disease (Ruda et al., 2024). Both agents were compared in a randomized phase 1 perioperative clinical trial of predominantly grade 2 IDH mutant glioma patients but included 6 patients with grade 3 tumors. Although, both drugs were found to be safe, vorasidenib had a better tumor penetration and was selected for the subsequent INDIGO clinical trial (Mellinghoff et al., 2023). INDIGO was a decisive phase 3, double-blinded trial which investigated the role of vorasidenib in adults with residual or recurrent grade 2 IDH mutant gliomas (Mellinghoff et al., 2023). Of 331 patients with no prior therapy other than surgery, 168 were randomized to receive vorasidenib 40 mg once a day. Patients randomized to the placebo arm were allowed to crossover to vorasidenib upon disease progression. At a median follow-up of 14.2 months, the primary endpoint of imaging-based PFS was significantly better for the vorasidenib group compared to the placebo group (hazard ratio 0.39, median 27.7 vs. 11.1 months, *p* < 0.001). The key secondary endpoint of time to next intervention was significantly improved in the vorasidenib group versus the placebo group (hazard ratio 0.26, p < 0.001). The safety profile of vorasidenib revealed low-grade toxicities with low numbers of serious adverse events and treatment discontinuations. INDIGO trial established a new treatment paradigm for a disease with largely “watch and wait” strategy, offering patients molecularly targeted treatment option. However, it is unclear if the PFS advantage will impact the OS as it will take years to tease out this information. The survival data will also be difficult to interpret due to the crossover design of the trial and as many as 32% of patients crossing over from the placebo arm to the vorasidenib arm. It is also unclear how efficacious vorasidenib is compared to the standard care of RT + PCV or RT + TMZ.

Newer IDH 1 inhibitors provide long-term responses in enhancing tumors and HGG in contrast to vorasidenib and ivosidenib (Ruda et al., 2024). D-2-HG inhibition and antitumor activity were seen in phase 1 clinical trials of IDH1 inhibitors Bay 1,436,032 and DS-1001 (Wick et al., 2021; Natsume et al., 2023). IDH1 inhibitor olutasidenib (FT-2102) was investigated in a nonrandomized phase 1b/2 clinical trial enrolling patients with relapsed or refractory IDH1 mutant glioma, 66% patients of which had HGG. This patient cohort was heavily pretreated with all patients receiving RT and 92% systemic therapy and surgery. Olutasidenib was well tolerated and showed antitumor activity with a 12-month PFS of 20.8% (de la Fuente et al., 2023). IDH1/2 inhibitors LY3410738 and HMPL-306 are being investigated in patients with IDH mutant solid tumors and gliomas (NCT, 2021, 2020).

Overall, IDH inhibitors have shown promising results with INDIGO trial results leading to the Food and Drug Administration (FDA) approval of vorasidenib in August 2024 for 12 years of older patients with grade 2 IDH1/2 mutant glioma following surgery. This is the first targeted therapy approved for IDH mutant glioma since the discovery of IDH mutations in 2008. Further clinical trials elucidating activity of IDH inhibitors either alone or in combination with chemoradiotherapy or other targeted therapy are necessary with a potential to advance survival and quality of life for patients with IDH mutant gliomas.

#### 7.4.2 Vaccine therapy

IDH1 R132H are frequent driver mutations encoding a shared clonal neopeptide recognized by mutation-specific T-helper cells. IDH1 R132H is expressed only in tumor and not in normal cells. These properties make IDH1 R132H an ideal candidate for a cancer vaccine target (Platten et al., 2021). An IDH1 R132H specific peptide vaccine was evaluated in a phase 1 clinical trial and was administered to 33 patients with grade 3/4 IDH mutant astrocytoma. It was found to be safe with adverse events restricted to grade. The vaccine administration was associated with a high number of pseudoprogressions. 3-year PFS was 64%, OS was 84% and immunity was induced in 93.3% patients (Platten et al., 2021). The successor trial, AMPLIFY-NEOVAC, was recently completed and randomized patients with relapsed IDH1 R132H positive glioma to receive immune checkpoint inhibitor (ICI) avelumab alone, IDH-vac alone or the combination of both. This was the first ICI clinical trial in the neoadjuvant setting and aims to comprehensively assess biologic responses to peptide vaccine and/or ICI (Bunse et al., 2022).

#### 7.4.3 Correction of epigenetic dysregulation

High levels D-2-HG result in epigenetic dysregulation leading to tumorigenesis and this is a potential pathway to exploit for the treatment of IDH mutant tumors. 5-azacytidine and decitabine are DNA methyltransferase inhibitors which induce DNA hypomethylation. This translates to reactivation of tumor suppressor genes and downregulation of oncogene expression. This mechanism as a potential option for therapy is being investigated in the preclinical and clinical settings (Alshiekh Nasany and de la Fuente, 2023).

#### 7.4.4 Poly(adenosine diphosphate ribose) polymerase (PARP) inhibitors

IDH mutant cells escape radiation and chemotherapy induced injury through PARP mediated DNA repair (Sulkowski et al., 2017). The OLAGLI trial showed some activity of olaparib monotherapy (Ducray et al., 2021); however, veliparib in combination with RT and TMZ failed to improve outcome in pediatric patients with IDH mutant gliomas (Karajannis et al., 2025). PARP inhibitors such as niraparib (NCT, 2021), olaparib (Ducray et al., 2021; NCT, 2019b), BGB290 (pamiparib) (NCT, 2019c) are being investigated as single agents or in combination.

#### 7.4.5 Immune checkpoint inhibitor (ICI) therapy

D-2-HG induced DNA hypermethylation silences programmed cell death-1 (PD-1) and its ligand (PDL-1) and this presents as a potential therapeutic option for patients with IDH mutant gliomas (Wang et al., 2016). Pembrolizumab and nivolumab are being investigated in combination with IDH inhibitors for patients with IDH mutant solid tumors including HGG (NCT, 2021, 2022, 2019).

## 8 Survival

### 8.1 IDH mutant astrocytoma

An inverse correlation is observed between WHO CNS grade and 1-year OS in patients with IDH mutant astrocytoma: 98, 92.4 and 76.3% for grades 2, 3 and 4, respectively. In patients with grade 3 and 4 tumours, 1-year OS is 95.7 and 87.5% with chemoradiotherapy compared to 69.5 and 32.3% without adjuvant therapy (Ostrom et al., 2023).

### 8.2 IDH mutant oligodendroglioma

1-year OS in IDH-mutant oligodendroglioma is generally high but shows a modest decline with increasing tumor grade: 97.9% in grade 2 compared to 94.4% in grade 3. Grade 3 patients have a better survival with adjuvant therapy: 1-year OS 96.8% with chemoradiotherapy and 68.3% without (Ostrom et al., 2023). Grade 3- median survival >14 years (van den Bent et al., 2017).

## 9 Prognosis

### 9.1 Tumor grade

Lower WHO tumor grades are associated with better survival in both IDH mutant astrocytomas and oligodendrogliomas. Median OS for grade 3 tumors is 8.1 years compared to 4.7 years for grade 4 tumors (Weller et al., 2024; von Deimling et al., 2018).

### 9.2 Pathological features

A recent reappraisal of WHO 3 oligodendroglioma patients from the French POLA cohort stratified patients into 3 groups with prognostic significance based on the presence of angiogenesis, mitoses and necrosis. Angiogenesis was detected with histology by identifying MVP and on imaging by seeing contrast enhancement (CE). Group 1 CE- included patients with high mitotic count only and no CE, Group angiogenesis patients displayed angiogenesis by MRI only (Group CE+) and/or by histopathology (MVP) but no necrosis (previous POLA Group 2) and Group 3 patients had MVP and necrosis. 10-year PFS for Group 1 CE-, Group angiogenesis and Group 3 were 73, 46 and 27%, respectively, (Figarella-Branger et al., 2025).

A histology panel review of 455 IDH mutant astrocytomas from 2 European Organization for Research and Treatment of Cancer (EORTC) clinical trials identified the mitotic index, using a cutoff of 2 mitoses per 10 high-power fields, as an independent prognostic marker in tumors lacking homozygous CDKN2A/B deletion. Neither necrosis (excluded due to low prevalence) nor MVP were found to be independent prognostic factors in this study (Kros et al., 2023).

### 9.3 CDKN2A/B loss and focal amplifications

#### 9.3.1 High grade astrocytoma

**Figure 7:**
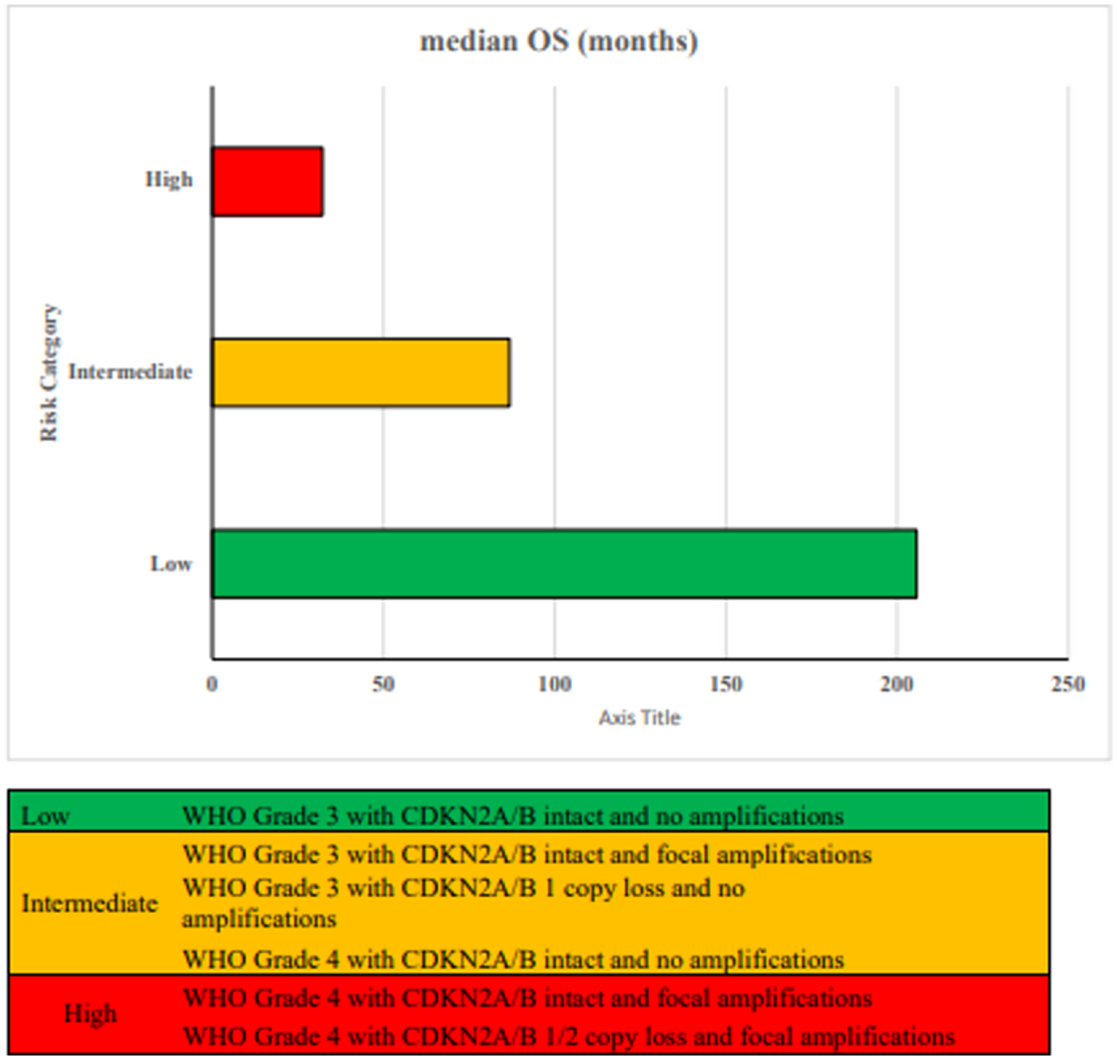
Proposed risk stratification based on WHO tumor grade, CDKN2A/B status and focal amplifications (Ghosh et al., 2025).

Incorporating CDKN2A/B status and gene amplifications with histopathologic grade improves the accuracy of overall survival prediction (Ghosh et al., 2025). Both homozygous and hemizygous losses of CDKN2A/B, as well as focal gene amplifications, are linked to poorer overall survival, independent of tumor grade (Korshunov et al., 2019). Three prognostically distinct groups are identified based on CDKN2A/B loss, focal gene amplifications and WHO tumor grade as shown in Figure 7.

Low risk patients with grade 3 tumors with intact CDKN2A/B and no focal amplifications have the best outcome with median OS of 205.7 months. Intermediate risk patients with grade 3 astrocytomas and CDKN2A/B hemizygous loss, intact CDKN2A/B but focal amplifications and grade 4 patients with intact *CDKN2A/B* and no focal amplifications have median OS of 80.4, 88.7 and 91.5 months, respectively. High risk patients with grade 4 tumors with intact CDKN2A/B and focal amplifications (55.9 months) or with loss of CDKN2A/B (hemizygous 31.9 months, homozygous 32.5 months) have the worst median OS (Ghosh et al., 2025; Weller et al., 2024; von Deimling et al., 2018). These results need further validation as another recent study of 334 patients with Grade 2/3 IDH mutant glioma showed no prognostic association of hemizygous CDKN2A/B deletions (Ippen et al., 2025).

#### 9.3.2 Grade 3 Oligodendroglioma

CDKN2A homozygous loss is associated with adverse prognosis in grade 3 oligodendroglioma (Figarella-Branger et al., 2025). In the French POLA cohort, the prognosis of such patients was close to or even worse than that of Grade 4 IDH mutant astrocytoma. In view of this dismal outcome, it is suggested to group these patients as Grade 4 oligodendroglioma (Appay et al., 2019).

### 9.4 DNA methylation and HOX genes

Through multi-platform analysis integrating methylation, gene expression, and somatic mutation, biomarker signatures of outcome have been characterized. Patients with low methylation and low transcription levels at HOX gene loci have the best outcome. Hox gene hypermethylation and overexpression was associated with worse survival. 7 HOX genes were sufficient to establish a poor signature outcome in both IDH mutant astrocytoma and oligodendroglioma (Mamatjan et al., 2023).

### 9.5 MGMT promoter methylation status

MGMT Promoter Methylation is associated with better OS and PFS for IDH mutant grade 4 astrocytoma only. With TMZ patients with methylated grade 4 astrocytoma had a median PFS/OS of 18.37–23.4/26.4–41.61 months compared to 6.97–9.33/9.1–13.76 months for unmethylated tumors (Chai et al., 2021; Yang et al., 2015).

## 10 Need for new treatment approaches for pediatric and AYA population

The current landscape underscores the need for more effective therapeutic strategies for younger patients. There is a distinction between pediatric IDH1/2 mutant LGG and MAPK-driven pediatric LGG, both biologically and clinically. Notably, similar to adult cases, pediatric IDH1/2 mutant gliomas exhibit a propensity for progression to higher histologic grades, accompanied by a correspondingly poor clinical prognosis. These observations suggest a potential rationale for the integration of adult treatment strategies, including more intensive therapeutic approaches, particularly at the time of disease recurrence (Yeo et al., 2023). Therapy-resistant tumor subclones are established early during gliomagenesis and contribute to tumor repopulation, with standard therapies exerting minimal selective pressure on the evolution of key genomic drivers in recurrent high-grade gliomas. Tumors exhibiting subclonal selection over time are associated with reduced patient survival, highlighting the potential benefit of early identification of aggressive subclonal populations to inform more effective, targeted therapeutic strategies (Fortin Ensign et al., 2023).

There is a temporal window in younger patients during which IDH mutant gliomas exhibit slower progression; however, once malignant transformation occurs, the disease rapidly adopts a more aggressive clinical course (Bennett et al., 2025). AYA population may be at increased risk for tumor progression and malignant transformation underscoring the importance of conducting AYA-specific clinical trials. Treatment strategies in this population require careful consideration, given the potential for prolonged survival and the necessity to balance therapeutic efficacy with the risk of long-term treatment-related sequelae. The development of targeted therapies against mutant IDH1/IDH2 may offer opportunities to defer or reduce the intensity of cytotoxic interventions, potentially mitigating the risk of both early treatment-related toxicity and malignant progression (Lim-Fat et al., 2024). Most of the completed or ongoing clinical trials for patients with IDH mutant HGG are adult-centric and exclude pediatric patients leading to a therapy gap for this population. CONNECT TarGeT D (NCT, 2023), part of a larger TarGeT umbrella trial, is a phase 2 clinical trial bridging this gap. This trial is investigating the efficacy of olutasidenib, an IDH1 inhibitor, along with RT and TMZ in patients with newly diagnosed HGG harboring IDH1 mutations (NCT06161974). The establishment of similar prospective cohorts with comprehensive molecular and clinical annotation will be critical to advancing our understanding of IDH mutant gliomas across the age spectrum. Future studies focusing specifically on AYA patients—incorporating survival outcomes alongside quality-of-life measures, patient-reported outcomes, and integrated molecular and imaging data—are essential to inform the development of optimized, age-appropriate therapeutic approaches for this distinct clinical subgroup.
